# Central venous catheter insertion- guidewire migration ratio: Right heart to inferior vena cava

**DOI:** 10.1371/journal.pone.0252726

**Published:** 2021-06-16

**Authors:** Daphna Reichmann-Ariel, Re’em Sadeh, Ori Galante, Yaniv Almog, Lior Fuchs

**Affiliations:** 1 Faculty of Health Sciences, Ben-Gurion University of the Negev, Be’er Sheva, Israel; 2 Clinical Research Center, Soroka University Medical Center, Be’er Sheva, Israel; 3 Medical Intensive Care Unit, Soroka University Medical Center, Be’er Sheva, Israel; Ohio State University Wexner Medical Center Department of Surgery, UNITED STATES

## Abstract

**Background:**

Central Venous Catheters (CVC) are being used in both intensive care units and general wards for multiple purposes. A previous study Galante et al. (2017) observed that during CVC insertion through Subclavian Vein (SCV) or the Internal Jugular Vein (IJV) the guidewire is sometimes advanced to the Inferior Vena Cava (IVC), and at other times to the right atrium. The rate of IVC wire cannulation and the association with side and point of insertion is unknown.

**Objective:**

In this study, we describe guidewire migration location during real time CVC cannulation (right atrium versus IVC) and report the association between the insertion site and side of the CVC and the location of guidewire migration, Right Atrium (RA)/Right Ventricle (RV) versus IVC guidewire migration.

**Methods:**

This is a retrospective study in the medical intensive care unit among patients that have received CVC during the study years 2014–2020. The rate of IVC versus right atrium/right ventricle wire migration during the procedure were analyzed. The association between the side and point of CVC insertion and the wire migration site was analyzed as well.

**Results:**

One hundred and sixty-six patients were enrolled. 33.7% of wires migrated to the IVC and 66.3% to the versus right atrium/right ventricle. The rate of wire migration to the IVC was similar in the IJV site and the SCV site. There was no association between the side of CVC insertion and wire migration to the IVC.

**Conclusion:**

About a third of all wire migrations, during CVC Seldinger technique insertion, were identified in the IVC, with no potential for wire associated arrhythmia. There was no association between CVC insertion point (SCV versus IJV) nor the side of insertion and the site of guidewire migration.

## Introduction

Central venous catheters are being used in both intensive care units and general wards for multiple purposes. During CVC cannulation, some complications may occur. One of those complications is extra systole or arrhythmia that can be caused due to irritation of the endocardium by the metallic guidewire, especially if the guidewire is inserted to an excessive depth [1, 2]. This phenomenon is not rare. A previous study [3] found that 41% of procedures resulted in atrial arrhythmias, and 25% produced some degree of ventricular ectopy.

Most arrhythmias resolve spontaneously or when the guidewire is withdrawn to an adequate depth [1]. Studies have shown that the most common arrhythmia associated with guidewire insertion is atrial premature contraction [1, 4]. However, there is also evidence of potentially life threatening arrhythmias such as persistent supra ventricular tachycardia [5], complete heart block [6] and sustained ventricular tachycardia that subsequent cardiac arrest [7] following the procedure. Wire irritation to the endocardium should be avoided to prevent these rare but dangerous complications.

The risk factors for developing an arrhythmia include insertion of wire longer than 20cm [1], female gender and patient height of under 170cm [4], and CVC insertion from the right subclavian position [3]. These factors indicate that the higher chances of tip entering right atrium or ventricle, the greater the chance of arrhythmia.

Guidelines for CVC insertion specify methods for verifying venous guidewire cannulation before dilation of the vein. Methods for confirming that the wire resides in the vein include, but are not limited to trans- esophageal echocardiography (identification of the wire in the superior vena cava or right atrium), continuous electrocardiography (identification of narrow-complex ectopy), or fluoroscopy [8]. None of these methods are routinely practiced, and performers insert the guidewire to a random length, sometimes causing short extrasystole (when the wire touches the endocardium) as verification for venous wire cannulation.

There is wide variation in distance from skin puncture site (internal jugular versus subclavian) and side of puncture (right versus left) and the right atrial-cava junction. One study measured these distance variations and found 12cm-25 cm gap [9]. There is no “one size fits all” distance to avoid endocardial irritation during CVC wire insertion.

A previous study [10], by our group describing the utilization of point of care ultrasound for CVC tip placement, observed that during CVC insertion through the subclavian vein or the internal jugular vein the guidewire may sometimes advance to the IVC, and in other times to the right atrium. When the guidewire went to the IVC there were no extra-systoles.

In this study, we aimed to describe guidewire migration location during real time CVC cannulation (right atrium versus IVC), and to report the association between the insertion site and side of the CVC (internal jugular versus subclavian, right side versus left side) and the location of guidewire migration, Right Atrium(RA)/Right Ventricle (RV) versus IVC guidewire migration.

The importance of this finding is related to the fact that guidewire migration to the IVC will not result in arrhythmia or an extrasystole, whereas guidewire migration to the right heart has the potential for arrhythmia.

## Material and methods

### Study population

This cohort study included one hundred and sixty-six patients admitted between 2014 and 2020 in the ICU in Soroka University Medical Center who required upper torso CVC (SCV or IJV) using Seldinger guidewire insertion and had full documentation of the procedure including the site of tip wire migration.

Patients were excluded if they had recent abdominal surgery precluding the subcostal view or if real time point of care ultrasound guidewire verification could not be made, or that the guidewire location was unknown.

This is a retrospective study of the prospectively and systematically collected data on CVC insertion under real time transthoracic ultrasound. The data was collected from the medical ICU in the Soroka medical center database of documented central lines inserted during the study years. Many of these central lines were inserted under real time ultrasound guidance with documentation of the guidewire migration site during the procedure. This was done as part of the protocol of central line insertion that was developed in this ICU^6^ and is part of the routine of CVC insertion.

We documented the date, the site and side of insertion, and the location where the guidewire tip was identified in the right heart or the IVC. Also, in random cases, we have documented if there were extrasystole or arrhythmias noticed on the monitor. Our research team accessed the databases to obtain the data during June 2020.

### CVC procedure

All catheters were inserted percutaneously using the Seldinger technique. It was inserted on one of four sites: The right internal jugular vein, The left internal jugular vein, The right subclavian vein, and the left subclavian vein. During the procedure we monitored the patients for arrhythmias. When the procedure was over, the guidewire’s location was verified using a trans thoracic US in the subcostal view, it was documented, and the CVC was sutured to the skin and covered sterilely.

### Use of US

There was a use of cardiac US during the procedure to identify the location of the tip of the guidewire inserted to the right heart or IVC in real-time, and to measure the depth of the guidewire when the tip of the guidewire is in the SVC. This is the method that we use, as the standard of care, for CVC insertion under US guidance for proper CVC tip placement as further explained in our previous study [10].

The subcostal view is used while inserting the guidewire to the central vein. Using the US, we look for the tip of the guidewire in the heart. Then we document if the guidewire went to the IVC, to the RA, or RV. The guidewire will be pulled back until the tip of the guidewire is seated in the SVC. Then the depth of the wire from the skin to the SVC is measured and documented, this is the depth that the central line will be inserted into.

### Ethics

This study was approved by the Helsinki committee of the Soroka Medical Center, Beer Sheva, Israel (Chairperson Prof R. Hershkovitz) on 12 July 2018. The ethics committee waived the requirement for informed consent.

### Statistical analysis

Patient characteristics are presented as mean ± SD for continuous variables with a normal distribution and categorical variable are presented as frequency (%).

In our univariable tests we compared means using the independent-samples t-test and compared proportions using Chi square test and Fisher exact test.

To examine the guidewire migration (IVC vs RA/RV) a logistic regression was conducted with the insertion site being the primary independent variable. Variables having clinical significance were add to the regression. An odds ratio (OR(, P-value and confidence interval was reported.

A two-sided P-value <0.05 was considered statistically significant for all statistical tests. P-values reported was rounded to two decimal places. All statistical analyses were performed using SPSS version 24.0 (IBM Corp, 2015).

## Results

**Fig 1:**
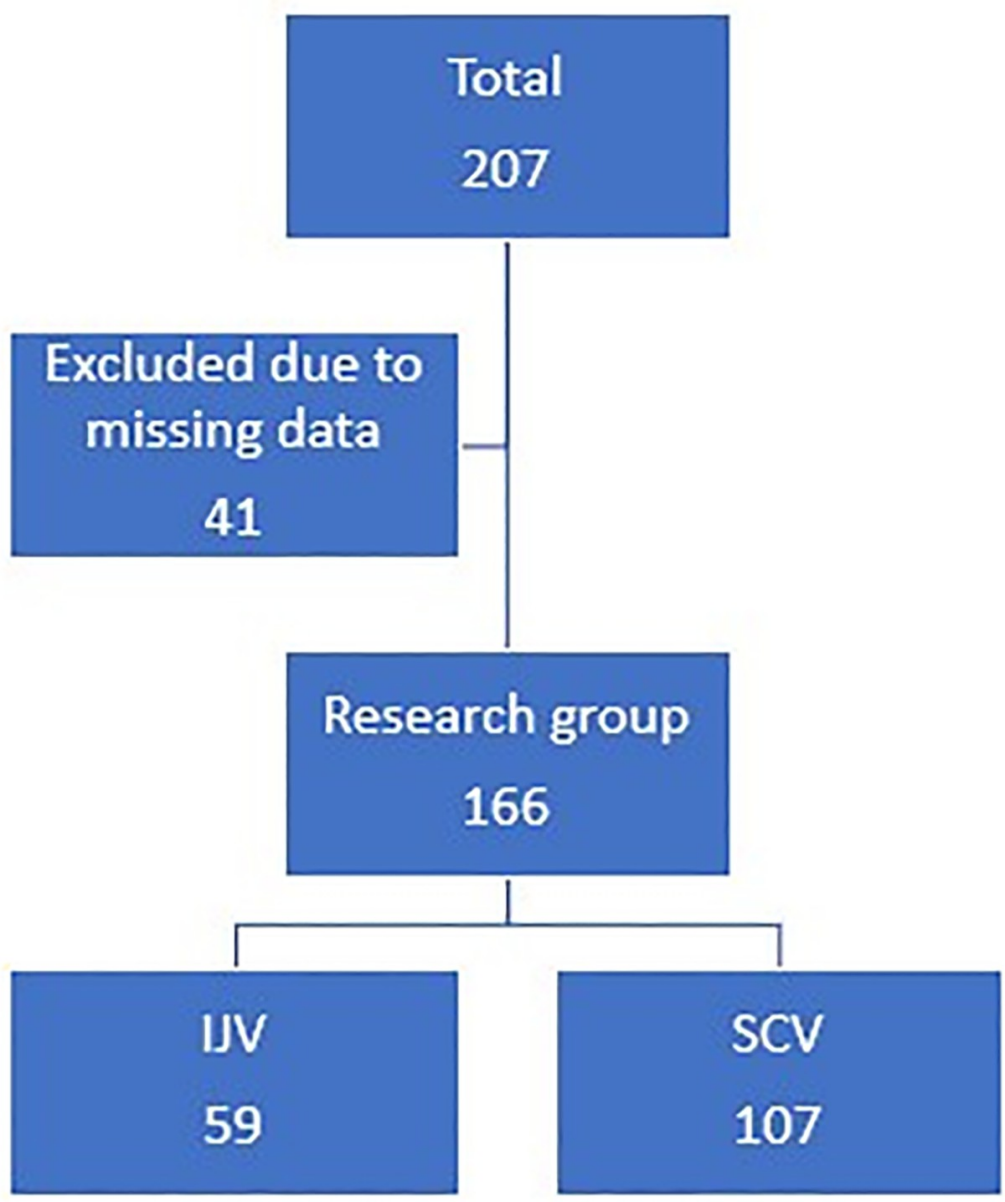
Research group.

Two hundred and seven patients were admitted to the medical ICU in Soroka University Medical Center in the years 2014–2020 who have required upper torso CVC insertion. Forty-one were excluded from this study due to missing data (see Fig 1). One hundred and sixty-six CVC cannulations from the IJV or the SCV were conducted and documented in this study. There were no differences in most of baseline characteristics between the two groups (Table 1). Table 2 outlines the characteristics of the patients in the study.

**Table 1:** Patients baseline characteristics.

	IJV	SCV	p-value
	N = 59	N = 107	
**Demographic Characteristics**			
Gender, male N (%)	40 (69%)	78 (73.6%)	0.52
Age, mean ± SD	55.3 ± 17.09	54.3 ± 19.1	0.72
BMI>30, N (%)	15 (31.3%)	31 (34.1%)	0.73
Smoking, yes N (%)	12 (22.6%)	31 (30.4%)	0.30
**Co- morbidities**			
CKD	7 (11.6%)	6 (5.6%)	0.15
HF	8 (13.6%)	11 (10.3%)	0.52
CV disease, yes N (%)	0	11 (10.3%)	**0.008**
Hyperlipidemia, yes N (%)	14 (23.7%)	28 (26.2%)	0.72
Anemia, yes N (%)	7 (11.9%)	3 (2.8%)	**0.019**
DM, yes N (%)	15 (24.5%)	27 (25.2%)	0.97
HTN, yes N (%)	27 (45.8%)	43 (40.2%)	0.48
COPD, yes N (%)	8 (13.6%)	9 (8.4%)	0.29
Arrythmias, yes N (%)	6 (10.2%)	15 (14%)	0.47

**Table 2:** Site and side of CVC insertion and wire migration site.

Total		166
**Tip placement**	**IVC**	56 (33.7%)
	**RA\RV**	110 (66.3%)
**Site insertion**	**IJV**	59 (35.5%)
	**SCV**	107 (64.5%)
**Side of insertion**	**Right**	112 (68.7%)
	**Left**	52 (31.3%)

Two thirds of upper torso CVC were inserted to the subclavian vein (64%) and a third to the internal jugular vein. About two thirds of CVCs were inserted from the right side (68.7%). About two thirds of wires migrated to the RA/RV and a third to the IVC (Table 2).

Although we had no systematic data collection of arrhythmias or extrasystoles documentation during CVC insertion, all 15 documented arrhythmias occurred when the wire migrated to the RA/RV and not when IVC wire migration was documented.

The site of CVC insertion was not associated with IVC or RA/RV wire migration (33.9% versus 33.6, IJV and SCV respectively, P = 0.97, Table 3A). The side of CVC insertion was also not associated with IVC or RA/RV wire migration (P = 0.22 Table 3B), nor the side and site together (P = 0.66, Table 3C).

**Table 3:** CVC insertion site and side.

**A**			
	**IJV**	**SCV**	**p-value**
	**N = 59**	**N = 107**	
Wire migration site, IVC N (%)	20 (33.9%)	36 (33.6%)	0.97
Side of insertion, right N (%)	39 (66.1%)	75 (70.1%)	0.59
**B**			
	**Right**	**Left**	**p-value**
	**N = 114**	**N = 52**	
CVC tip placement, IVC N (%)	35 (30.7%)	21 (40.4%)	0.22
**C**			
	**IJV**	**SCV**	**p-value**
	**N = 39**	**N = 75**	
CVC tip placement, IVC N (%)	13 (33.3%)	22 (29.3%)	0.66

## Discussion

We have found that during the insertion of CVC using the Seldinger technique, 33% of guidewires migrate to the IVC, with no potential for any guidewire induced arrhythmia. We also found no association between site and side of CVC insertion and the site of guidewire migration; hence choosing the subclavian over the internal jugular on any side will not increase the rate of guidewire migration to the IVC or the RA/RV.

We believe that physicians who perform central venous catheter insertion should be familiar with the relatively high IVC guidewire migration prevalence.

CVC are being used widely in the medical world for different purposes outside of the ICU, in perioperative care, temporary and long-term dialysis access, long term access for chemotherapy or parental nutrition and many other uses. Although we did not measure the applicability of wire migration in other settings, we believe that our findings are applicable to all CVC inserted using the Seldinger technique.

Operators may confirm CVC positioning when extrasystole occurs and may feel uncertain when there is none. This technique should not be encouraged for wire placement confirmation because of the risk for right heart guidewire migration hence the potential risk for life-threatening arrhythmia and low sensitivity for such tachycardia. We show that over 30% of cases will not have the potential for tachyarrhythmia. Also, Performers should know that choosing a specific site of insertion (SCV versus IJV) or specific side (right versus left) will not increase or reduce the chance for RA/RV guidewire migration, hence, the chance for iatrogenic arrhythmia.

Our novel technique of CVC insertion enables us to locate the tip wire using POCUS. As we have described in a previous study [10], by using this technique, we reduce the need to reposition the line in a two-step procedure that can increase the risk of infection and other complications, and we can significantly decrease the time to line utilization.

There are a few possible limitations to this study. First, all the patients in the study were treated in the same medical ICU in Soroka medical center and by a limited number of senior physicians and residents, limiting the generalizability. Secondly, the real time POCUS CVC insertion technique was first developed in the ICU during the early study years. At first it was not used in all CVC procedures, and therefore in that time only a small number of the CVC procedure performed in the ICU were documented in the database. This may hold potential selection bias, but we believe that the missing data was mainly from the early years of data acquisition, while in the latter years most CVC insertions were documented, including documentation of cases where we failed to find that tip wire inside the heart. We believe that this will not affect the primary measured outcome of tip wire migration site. Thirdly, extrasystole was not routinely documented as part of the protocol, so although the documented ones occurred only when the wire was identified in the RA/RV, we did not design the study to prove this observation. Lastly, only 20 patients received left-sided IJV CVC. This limited sample size may influence the results as well.

## Conclusions

Clinicians who perform upper torso CVC insertion should be aware that 33% of guidewires migrates to the IVC, and not to the RA/RV. When a guidewire migrates to the IVC, no extrasystole is observed.

There was no association between the site of insertion, side of insertion, and the site of guidewire migration. Tachycardia on the monitor should not be used as a sign for correct placement of the central line during this procedure.

## Supporting information

S1 File (ZIP)

S1 Data (XLSX)
