# Supplementary figures and images for: Central venous catheter insertion- guidewire migration ratio: Right heart to inferior vena cava
Source: PLoS One. 2021 Jun 16;16(6):e0252726. doi: 10.1371/journal.pone.0252726 (PMC8208537; doi:10.1371/journal.pone.0252726)

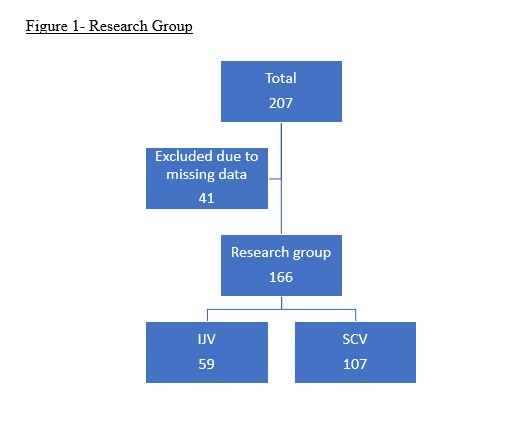

Supplement: S1 File — (ZIP) [file pone.0252726.s001.zip › Fig1.tif]

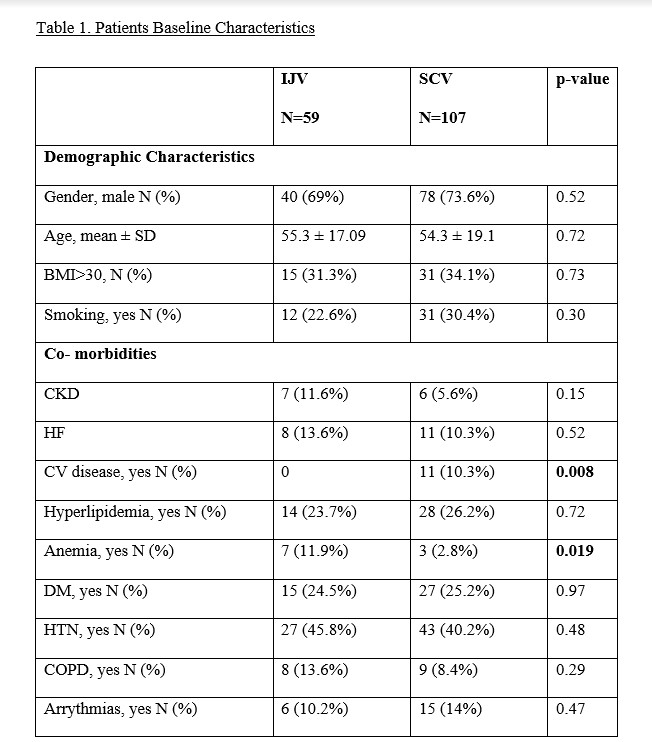

Supplement: S1 File — (ZIP) [file pone.0252726.s001.zip › Table1.tif]

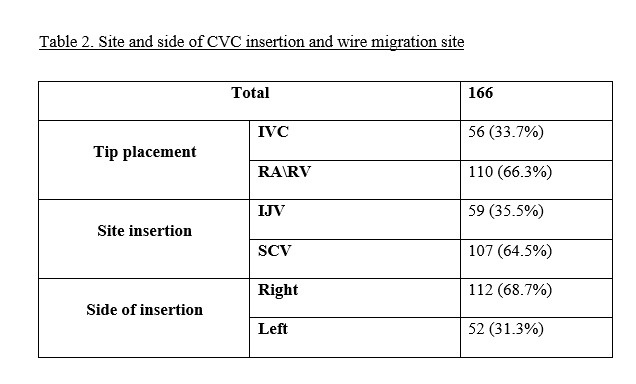

Supplement: S1 File — (ZIP) [file pone.0252726.s001.zip › Table2.tif]

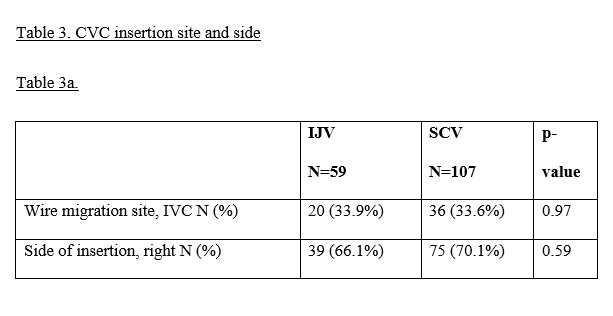

Supplement: S1 File — (ZIP) [file pone.0252726.s001.zip › Table3a.tif]

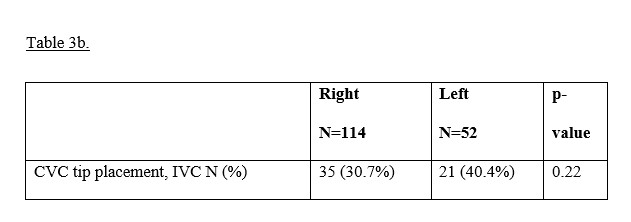

Supplement: S1 File — (ZIP) [file pone.0252726.s001.zip › Table3b.tif]

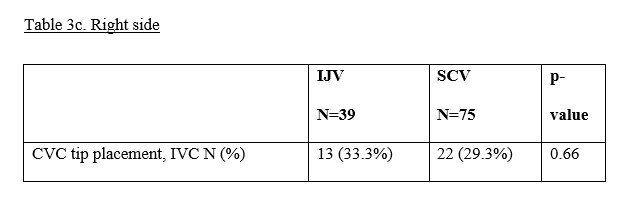

Supplement: S1 File — (ZIP) [file pone.0252726.s001.zip › Table3c.tif]
